# Involvement of the gene encoding the collagen type II alpha 1 chain in mandibular mobility

**DOI:** 10.1186/s12903-025-06245-2

**Published:** 2025-06-25

**Authors:** Bianca Cavalcante-Leão, João Armando Brancher, Fernanda Bertoli, Michelle Nascimento Meger, Luisa Helena Batista, Thais Vilalba, Maria Angélica Hueb de Menezes-Oliveira, Flares Baratto-Filho, Christian Kirschneck, Juliana Feltrin-Souza, Erika Calvano Küchler

**Affiliations:** 1 School of Dentistry, Tuiuti University of Paraná, Curitiba, Paraná Brazil; 2 Postgraduate Program in Cell and Molecular Biology, Federal University of Paraná, Curitiba, PR Brazil; 3 Department of Biomaterials, University of Uberaba - UNIUBE, Uberaba, Minas Gerais Brazil; 4 Department of Dentistry, Univille– University from the Joinville Region, Joinville, Santa Catarina Brazil; 5 Department of Orthodontics, University of Bonn, Medical Faculty, Welschnonnenstr. 17, Bonn, 53111 Germany; 6 Department of Stomatology, Federal University of Paraná, Curitiba, Paraná Brazil

**Keywords:** Mandibular movements, Collagen type II alpha 1 chain, Genetics

## Abstract

**Background:**

There are no studies investigating the role of COL2A1 genes for mandibular mobility. The aim of this study was investigate the association between mandible mobility with single nucleotide polymorphisms (SNPs) in gene encoding collagen type II alpha 1 chain (COL2A1).

**Methods:**

Adolescents age from 10 to 14 years old without restrictions in mandibular movement were evaluated. The mandibular mobility was recorded using rule. Genomic DNA was obtained from saliva and the SNPs in COL2A1 gene (rs1793953 and rs2276454) were genotyped by real time polymerase chain reactions. One-way ANOVA with Tukey’s post-test was used to compare the mean values of the mandibular mobility among genotypes in the co-dominant model. A linear regression analysis was also performed.

**Results:**

99 adolescents were included (53 boys and 46 girls). Gender and age were not associated with mandibular movements. For the rs1793953, unassisted opening, assisted opening and contralateral movements were statistically different among genotypes. The rs2276454 was associated with larger protrusive movements, in patients carrying TT genotype presented higher protrusion mean values than CT and TT individuals (*p* = 0.0046).

**Conclusions:**

Our findings suggest that genes may play an essential role for the normal variation in mandibular movement and SNPs rs1793953 and rs2276454 in *COL2A1* may explain mandibular mobility variations.

## Background

The masticatory system consists of a mandible which is able to move in relationship to the skull and is guided by two temporomandibular joints (TMJs) through contractions of the masticatory muscles and participation of bones and ligaments [1]. As the maxilla is static, mandibular movements of rotation, translation, opening, closing, protrusion, retrusion, and laterotrusion essentially depend on the relationship between the mandibular condyle, the articular disc and the articular surface of the temporal bone [2]. On both the right and left sides, the articular surfaces of the TMJ are separated into a lower compartment, which comprises the mandibular condyle and the inferior surface of the articular disc, and the upper compartment, represented by the glenoid fossa in the temporal bone and the superior surface of the articular disc. As TMJs support tensile and compressive forces, they contain fibrocartilage with collagen type I and II, essential for kinematic action between the right and left condyles during function [3].

Type II collagen is synthesized by chondrocytes and is an essential component of the extracellular matrix. Their secondary structure includes three identical alpha-1(triple helix) chains that maintains the stability of connective tissues and have a major importance for normal joints function [4, 5]. The gene encoding the type II collagen is Collagen Type II Alpha 1 chain (*COL2A1*) located on chromosome 12q13.11-13.2 [6]. The *COL2A1* gene consists of 54 exons, which encode 1487 amino acids [7, 8]. The majority of the variations in the *COL2A1* gene are simple nucleotide substitutions, called single nucleotide polymorphisms (SNPs). Interestingly, recent studies in humans have shown that SNPs in *COL2A1* are associated with orofacial phenotypes, including mandible size [9–12] and temporomandibular disorder [13].

The paired TMJ operate the mandible’s movement and allow the mandibular mobility [14]. The occurrence of particularly pronounced mandibular excursions is called hypermobility, which is a disorder of the human jaw joint. Hypermobility is defined by “greater than normal range of motion in a joint, which may occur naturally in otherwise normal persons or may be a sign of joint instability” [15]. Assessment of sagittal and vertical mandibular mobility is an integral part of many dental clinic examinations. There are no studies investigating the role of the genes for the mandibular mobility variation [16]. Therefore, the aim of present study was to investigate the association between the degree of the mandible mobility with SNPs in *COL2A1*.

## Methods

### Study participants

This cross-sectional study was approved by the Committee for Ethics in Research with Humans of Paraná Federal University (protocol number 2.006.086). Participants received written information about the study, and signed consent was obtained from a parent for all parents/guardians. The study followed the Declaration of Helsinki guidelines and was conceived according to the principles for reporting the results of genetic association studies defined by the Declaration of the Strengthening the Reporting of Genetic Association Studies (STREGA) [17]. The adolescents and legal guardians received information about the study, and informed consent was obtained from all participants.

Criteria to data collection from adolescents included in the sample was previously described [18], briefly a clinical exam was executed in the adolescents from September 2014 to July 2016. Adolescents aging 10 to 14 years old enrolled in private and public schools in the city of Curitiba-Brazil, were included. Adolescents who presented underling syndromes, facial trauma, facial pain of odontogenic or non-odontogenic origin, including temporomandibular disorder, patients with orthodontic appliances, myorelaxant bite plates or prostheses, severe facial anomalies, dental anomalies, dental caries lesions with extensive coronary destruction, severe periodontal problems, systemic disorders with cognitive or behavioral impairment, speech disorders, use of medications such as antidepressants, muscle relaxants, nonsteroidal anti-inflammatory drugs and severe psychiatric disorders were excluded.

### Mandibular mobility examination

The clinical examinations were conducted in the school environment, with the adolescents seated in chairs in front of a window to obtain the maximum natural lighting. The adolescents were asked to move the mandible, opening the mouth for maximum opening and after in a protrusive and lateral movement to the left and right. The mandibular mobility was recorded manually using a millimeter rule as follows:

i. Maximal opening movement unassisted: the adolescent was asked to open their mandible without any assistance from the examiner. The distance between the incisal edge of the maxillary right central incisor and mandibular right central incisor was registered with a millimeter ruler.
ii. Maximal opening movement assisted: in this situation the examiner helps the adolescent to extend their open mandible and as wide as possible. The distance between the incisal edge of the maxillary right central incisor and mandibular right central incisor was registered with a millimeter ruler.
iii. Maximal protrusive movement: the adolescent was asked to protrude the mandible as far as possible. The distance between the labial surface of the incisal edge of the maxillary right and the labial surface of the mandibular right central incisor was measured.
iv. Contralateral movements to the left and right (Laterotrusive movement): With the posterior teeth in maximal intercuspal position, a vertical line was drawn with a pencil between the mesial-labial surfaces of the maxillary and mandibular central incisors, i.e., midline axis and the adolescent move the mandible as wide as possible to the left and to the right, respectively. Lateral excursion measures were made between the two lines. Eventual discrepancies between the maxillary and mandibular midline axes were considered.

Overjet and Overbite measurements: for the overjet measurement, the vertical linear distance between the upper and lower incisal edges was measured at the height of the occlusal plane; the incisal overbite was carried out by measuring the distance between the lower and upper incisal edges measured perpendicular to the occlusal plane. If the adolescent presents an anterior open bite, the negative overbite value was subtracted from the value of the interincisal distance. All measurements were expressed in millimeters.

### DNA samples and genotyping and sample-size calculation

Saliva samples were collected from the adolescents randomly selected from the initial sample [19]. The sample-size calculation was performed based on the mean difference between two independent means of 1.0 (standard deviation of 1.3) and assuming the minor allele frequency of 0.40. Using the parameters of alpha = 5% and power = 80%, the calculation predicts a minimum of a total of 82 patients.

Genomic DNA for molecular analysis was obtained from epithelial cells of the oral mucosa using a mouthwash consisting of 5 ml of a 3% glucose solution in a falcon tube 15 ml and the DNA extraction followed a previously published protocol [20].

The DNA was quantified, and its purity was assessed by measuring 260/280 ratio, using a NanoDrop 2000 spectrophotometer.

SNPs in *COL2A1* gene (rs1793953 and rs2276454) (Table 1) were genotyped by real time polymerase chain reactions (PCR) using the TaqMan assay (StepOnePlus Real-Time PCR System, Applied Biosystems). Data analysis for allelic discrimination was carried out using the StepOnePlus Software. The SNPs were selected through the consultation of the International HapMap Project (www.hapmap.org) page, which is a union of several countries for the development of a map with patterns of variations of the DNA sequence.

**Table 1:** Studied SNPs in *COL2A1*

Position	SNPs	MAF globally	Change Base in the context
12q13.11	rs1793953	0.49	TCAGGCTC*[A/G]*TTCACTC
	rs2276454	0.43	TTACCCTGTC*[A/G]*CCTTTGGGC

Note: MAF mean minor allele frequency. Obtained from database: ncbi.nlm.nih.gov

### Statistical analysis

Data were analyzed using Graphpad Prism 9 (Graph-Pad). The chi-square test was used to calculate whether the SNPs were in Hardy-Weinberg equilibrium. Shapiro-Wilk was used to test normality of the data. Linear regression analysis including age, gender and genotypes were performed. A level of significance of 0.05 was used.

## Results

A total of 99 adolescents were included: 53 (53.5%) boys and 46 (46.5%) girls. The age ranged from 10 to 14 years old, and the mean age was 11.1 years (SD = 1.12). Age and gender were not associated with mandibular movements (Table 2).

Both studied SNPs were in Hardy-Weinberg equilibrium (chi-square for rs1793953 = 1.16 and chi-square for rs2276454 = 0.062). Means and standard deviations according to the genotypes are presented in the supplementary Table 1. The linear regression analysis results are presented in the Table 3.Genotypes in both SNPs were associated with the degree of the mandible mobility (*p* > 0.05).

**Table 2:** Mandibular movements comparison with age and gender

Phenotypes / Independent variables	SE	95% CI	*p*-value
*Maximal opening movement unassisted*			
Age	0.407	-0.436 to 1.181	0.363
Sex	0.911	-2.102 to 1.514	0.747
*Maximal opening movement assisted*			
Age	0.416	-0.394 to 1.258	0.301
Sex	0.930	-2.488 to 1.205	0.491
*Protrusive movement*			
Age	0.115	-0.052 to 0.402	0.131
Sex	0.256	-0.664 to 0.353	0.546
*Contralateral movement (right)*			
Age	0.146	-0.018 to 0.562	0.065
Sex	0.326	-1.177 to 0.119	0.108
*Contralateral movement (left)*			
Age	0.230	-0.063 to 0.523	0.122
Sex	-0.482	-1.137 to 0.173	0.147

Note: SE means standard error; CI means confidence interval

**Table 3:** Mandibular movements mean and standard deviations (SD) in millimetre according to the genotypes

**Phenotypes**	**Genotypes (mean SD)**			***p*** **-values**
**rs1793953**	**CC (n = 40)**	**CT (n = 41)**	**TT (n = 18)**	
Maximal opening movement unassisted	47.13 (4.42)^a^	50.51 (4.02)^b^	48.17 (4.36)^a^	< 0.0001*
Maximal opening movement assisted	49.35 (4.44)^a^	52.93 (4.14)^b^	49.72 (4.35)^a^	0.0007*
Protrusive movement	4.40 (1.3)	4.75 (1.24)	4.17 (1.58)	0.2095
Contralateral movement (right)	7.32 (1.70)^a^	8.36 (1.49)^b^	7.72 (1.56)^ab^	0.0153*
Contralateral movement (left)	7.37 (1.76)^a^	8.39 (1.44)^b^	7.55 (1.54)^ab^	0.0149*
**rs2276454**	**CC (n = 14)**	**CT (n = 48)**	**TT (n = 37)**	
Maximal opening movement unassisted	47.93 (6.09)	48.17 (4.60)	49.73 (3.47)	0.2200
Maximal opening movement assisted	50.86 (6.11)	50.15 (4.66)	51.89 (3.72)	0.2220
Protrusive movement	4.78 (1.67)^ab^	4.08 (0.89)^a^	4.94 (1.37)^b^	0.0046*
Contralateral movement (right)	6.93 (2.49)	8.04 (1.24)	7.89 (1.66)	0.0796
Contralateral movement (left)	7.14 (2.50)	7.94 (1.26)	7.94 (1.70)	0.2486

Note: ANOVA and Tukey’s post hoc tests. *the mean difference is significant at the level 0.05

## Discussion

In the past two decades, the genetic background of several craniofacial traits has been widely explored, and the molecular etiology of many dental-facial conditions has been unraveling. Temporomandibular disorder is one of the most studied traits in the craniofacial complex and is characterized by limitation and deviation in the trajectory of mandibular movements, limiting the mandibular kinematics [21]. So far, some reviews have pooled several candidate genes for temporomandibular disorder [22, 23], including genes involved in coding for condylar cartilage [10]. One of these genes is *COL2A1* [13], which is the gene encoding a fibrous protein whose chains form supramolecular aggregates that contribute to maintain the structural integrity of the extracellular matrix, the type II collagen [24]. This protein coats the articular surfaces of the TMJ and supports multi-vectorial forces [3]. The relationship between *COL2A1* and temporomandibular disorder have been investigated in in animal models and humans. A study using transgenic mice that expresses the deletion mutation of the Col2a1 gene (the human homologue of the *COL2A1*) were compared their normal siblings. The result demonstrated that the transgenic mice present defects in condylar cartilage early in life and progression of degenerative changes occurs until adult life [25]. Another study evaluating *Col2a1* mutant mice observed condylar cartilage abnormalities at early ages, in which *Col2a1* C-propeptide mutation showed disorganization of chondrocytes and abnormalities in proteoglycan development. These aspects seem to change articular cartilage integrity leading to a degradation [26]. Therefore, in the present study we evaluated if the SNPs rs1793953 and rs2276454 in COL2A1 have a role in mandibular mobility in humans and we observed that these SNPs may be involved in mandibular movement variations among individuals.

Although the genetic background of several craniofacial phenotypes has been extensively studied, to the best of our knowledge, this is the first study to investigate genes involved in mandibular mobility. Mandibular mobility disorders are not uncommon findings in humans [27, 28]. Clinically, symptomatic hypermobility of the mandibular joint is used as a mild subdivision of subluxations, in which patients often report clicking joint sounds and irregular movements of the mandible during wide opening and closing of the mouth [29]. It is important to emphasize that in our study, we evaluated the normal variation of mandibular movement in adolescents. In order to minimize the bias, we also exclude adolescents with any sign or symptom of temporomandibular disorder, once there is evidence that mandibular mobility in patients with temporomandibular disorder is affected [30].

Although mandibular mobility could present gender difference [27, 28], this trend was not observed in our results, in which boys and girls presented similar values for all the measurements performed. It is important to mention that this result could reflect the age of our sample. Hormonal differences in adolescents these ages may not play an important role yet in the mandibular condyle. However, the current literature clearly supports the role of sexual hormones on the temporomandibular disorder [31, 32] and temporomandibular joint [33–37].

Our study strongly suggested that both SNPs, rs1793953 and rs2276454, in *COL2A1*, are involved with mandibular mobility. It is of interest to note that the studied SNPs are intronic variants, however, some previous studies support their clinical relevance in different phenotypes. The rs1793953 was associated with the development of knee osteoarthritis [38]. The rs2276454 was associated with degenerative lumbar scoliosis [39]. Both SNPs (rs1793953 and rs2276454) were also associated with clinic pathological features of intervertebral disc degeneration [5].

Interestingly, rs2276454 was previously associated with disc displacement of the TMJ in this sample population [13], in our results this SNP was associated with protrusive movement and also left and right contralateral movements of the mandible. The rs1793953 was associated with almost all mandibular movements evaluated in our sample.

The knowledge regarding COL2A1 in mandibular mobility, for example, that certain genotypes exhibit greater maximal opening and lateral excursions means clinicians could anticipate and monitor patients who may be predisposed to excessive TMJ movement or early hypermobility. Once hypermobility of the mandible can predispose to TMJ disorders, pain, and degenerative changes over time. Genetic screening may help flag atrisk children and adolescent before clinical symptoms arise.

Briefly, our results bring new evidence that genes play an essential role in the normal variation in mandibular movement. One important limitation of our study is the gene coverage, in which only two SNPs were studied. Therefore, we suggested that future studies explore other SNPs in *COL2A1*, and other genes expressed in mandibular condylar cartilage. Understanding the molecular etiology of mandibular mobility in pathological conditions should also be explored in future research.

## Conclusion

Our findings suggest that genes may play an essential role for the normal variation in mandibular movement and SNPs rs1793953 and rs2276454 in *COL2A1* may explain mandibular mobility variations.

## Data Availability

The datasets supporting the conclusions of this article is publicly available in the figshare repository: https://doi.org/10.6084/m9.figshare.28451039.v1.
